# A Case of Neuromyelitis Optica Spectrum Disorder With Improvement in Urinary Retention After the Administration of Ravulizumab

**DOI:** 10.7759/cureus.75827

**Published:** 2024-12-16

**Authors:** Takuya Saito, Tatsuhito Ishii, Tsuyoshi Uchiyama, Keishiro Sato

**Affiliations:** 1 Neurology, Seirei Hamamatsu General Hospital, Hamamatsu, JPN

**Keywords:** immunotherapy, myelitis, neuromyelitis optica, urinary catheterizations, urinary retention

## Abstract

Neuromyelitis optica spectrum disorder (NMOSD) is an inflammatory disease that causes recurrent neuritis and myelitis. Ravulizumab, a complement protein C5 inhibitor, was developed to treat NMOSD. However, its efficacy in improving symptoms remains unclear. This case report describes the case of a 30-year-old woman with NMOSD who developed thoracic myelitis. Initial treatment with high-dose methylprednisolone and hemodialysis alleviated paraplegia, although urinary retention persisted. Two months after initiating ravulizumab treatment, urinary function improved. Ravulizumab administration may have contributed to the improved urinary retention.

## Introduction

Neuromyelitis optica spectrum disorder (NMOSD) is an autoimmune inflammatory disease that causes repeated attacks of neuritis and myelitis [1]. Myelitis caused by NMOSD is characterized by longitudinally extensive transverse myelitis [2] and often leads to serious sequelae after a single attack. One of the sequelae of myelitis is urinary retention [3]. Patients with urinary retention may require self-catheterization [4]. Ravulizumab has recently been developed as a biological agent for NMOSD [5]. Ravulizumab is used as a maintenance treatment for NMOSD [6]. However, its efficacy in improving the symptoms remains unclear. Herein, we report a case of thoracic myelitis due to NMOSD in which urinary retention, a sequela of myelitis, improved after the administration of ravulizumab.

## Case presentation

A 30-year-old woman developed encephalitis a year ago and was diagnosed with NMOSD with serum aquaporin-4 (AQP4) antibody positivity. The patient received an acute-phase treatment and did not experience any sequelae. She was prescribed 20 mg of prednisolone (PSL), which was gradually reduced to 10 mg.

**Figure 1:**
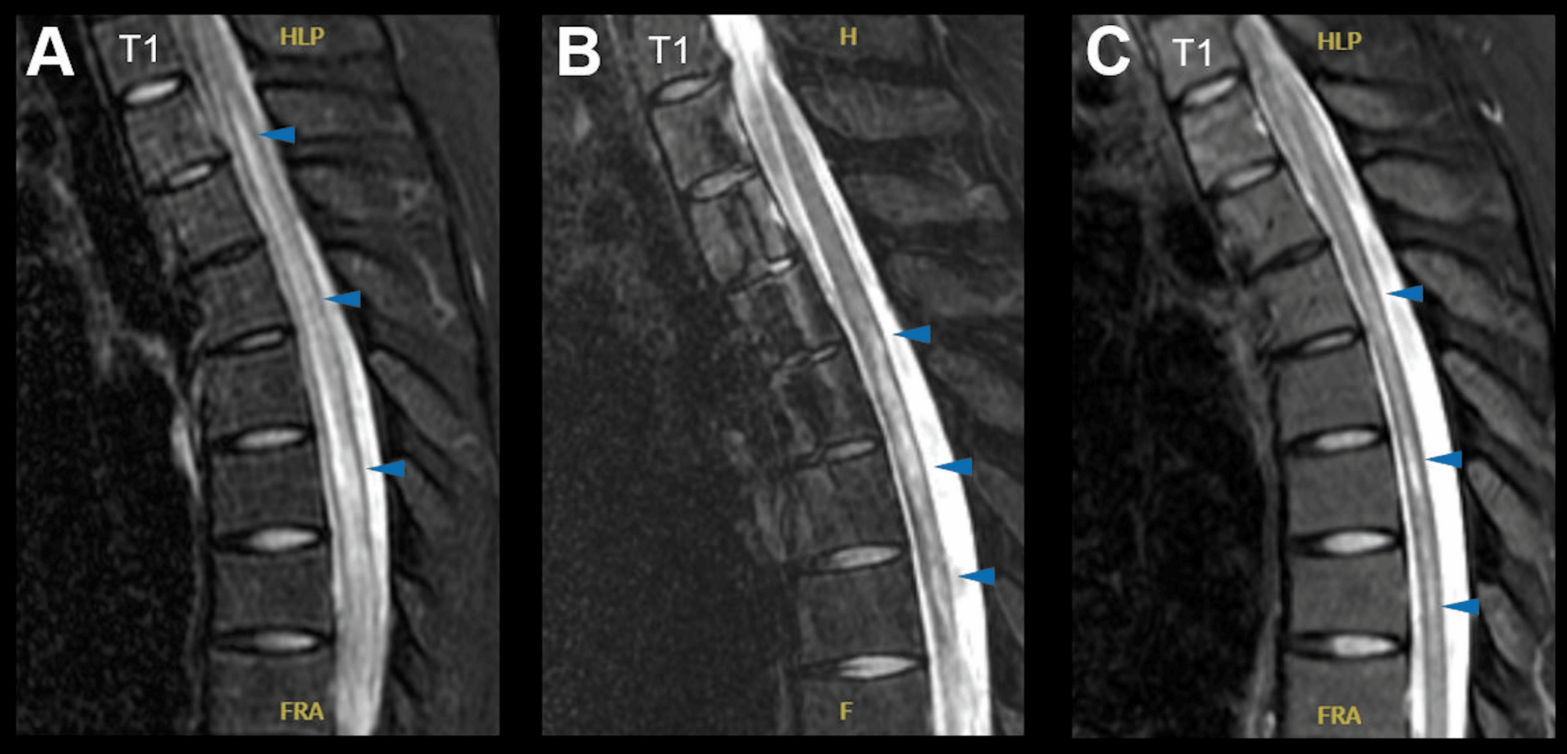
Magnetic resonance imaging findings. Longitudinally extensive thoracic transverse myelitis on T2-weighted sagittal magnetic resonance imaging at admission (A). The spinal cord lesions shrank, and swelling reduced after one month (B). The spinal cord lesions further reduced in size after nine months (C).

She noticed that she had difficulty urinating. Two weeks later, she visited our hospital because of urinary retention and difficulty walking. Physical examination revealed paraplegia, numbness, allodynia below the T4 spinal level, increased deep tendon reflexes in the lower extremities, bilateral Babinski reflex, and urinary retention. Blood and urine tests revealed no remarkable findings. Cerebrospinal fluid (CSF) examination revealed pleocytosis (32 cells/μL; 32 monocytes) and a slightly elevated protein level (56 mg/dL). Magnetic resonance imaging (MRI) showed T2-hyperintensities at the T2-T9 level (Figure 1A), and these lesions showed swelling and no enhanced effects. Brain MRI had no acute lesions. The patient was diagnosed with thoracic transverse myelitis secondary to NMOSD.

The patient was initially treated with high-dose intravenous methylprednisolone (1 g for two days and 500 mg for seven days) and immunoadsorption therapy. The patient underwent plasma exchange and immunoglobulin therapy as paraplegia worsened. The spinal cord lesions shrank, and swelling was reduced on MRI after acute treatment (Figure 1B). Her lower limb muscle strength and sensory impairment improved; however, she continued to have urinary retention and required urethral catheterization. After acute treatment, the patient was restarted on 20 mg PSL. She continued to self-catheterize intermittently because she was unable to urinate due to urinary retention.

Three months after developing myelitis, she was administered ravulizumab following the meningitis vaccine. The PSL dose was gradually reduced. The patient began to urinate on her own two months after the administration of ravulizumab and no longer needed to undergo self-catheterization during the day four months after the administration of ravulizumab. The spinal cord lesions were further reduced in size on MRI (Figure 1C).

## Discussion

Here, we report the case of a patient with NMOSD who developed thoracic myelitis. Urinary retention gradually improved after ravulizumab administration.

Urinary retention is a serious sequela of myelitis that greatly reduces the quality of life. Urinary retention that occurs in the acute phase often persists for a long time in patients with myelitis who develop long spinal cord lesions [7]. Even when other neurological symptoms of myelitis improve, urinary dysfunction persists [8]. In young women, continuing to perform intermittent catheterization is a great physical and mental burden. The improvement in urinary retention considerably improved their quality of life.

In NMOSD, extensive demyelination and inflammation affect multiple spinal cord segments, accompanied by astrocyte death, axonal loss, perivascular lymphocytic infiltration, and vascular proliferation [9]. AQP4 immunoglobulin G antibodies, the primary pathogenic factor in NMOSD, can cross the blood-brain barrier and bind to AQP4 in astrocyte end-feet, triggering complement recruitment and activation, which leads to complement-dependent cytotoxicity [10]. NMOSD causes more severe clinical symptoms than multiple sclerosis due to the massive inflammation of the spinal cord [11].

Ravulizumab is a monoclonal antibody directed against the complement protein C5 [5]. Patients receiving ravulizumab are at increased risk for meningococcal infection, as the terminal complement system plays a crucial role in protecting against meningococcal disease. All patients receiving ravulizumab must be vaccinated against meningococcal disease [12], as in this case. Ravulizumab significantly reduced the risk of relapse in patients with AQP4 antibody-positive NMOSD [6]. The possibility of improvement in neurological symptoms following acute treatment with ravulizumab has been reported [13,14]. Furthermore, chronic cognitive improvement following ravulizumab administration has been reported [15]. In the present case, urinary retention, which did not improve during the acute phase, improved after ravulizumab administration. Although the natural progression of urinary retention over a long period cannot be ruled out, the chronic anti-inflammatory effect of ravulizumab may have contributed to the improvement in urinary retention.

Because this is a single case report, the true reason for the long-term improvement in myelitic urinary retention cannot be determined. Therefore, further investigations are required.

## Conclusions

We encountered a case of thoracic myelitis due to NMOSD. Her urinary retention did not improve with acute treatment, although it did improve after ravulizumab administration in the chronic phase. Her quality of life greatly benefited from the improvement in her urinary retention. Ravulizumab administration may have contributed to improvements in urinary retention in NMOSD.
